# Annotation Marathon Validates 21,037 Human Genes

**DOI:** 10.1371/journal.pbio.0020166

**Published:** 2004-04-20

The announcement of the human genome sequence three years ago was widely hailed as one of the great scientific achievements in modern history, and with good reason. Determining the structure and nature of the genetic code promises to provide valuable insights into human evolution and the molecular basis of disease. But sequencing the genome is just the first step toward this decidedly worthy goal—the monumental task of ascribing biological meaning to those sequences has just begun. And while researchers know a great deal about some of the 30,000 or so genes in the human genome, they have yet to ascribe function to the majority of them. Takashi Gojobori and a large international team of collaborators have now taken a big step toward narrowing this knowledge gap.[Fig pbio-0020166-g001]

**Figure pbio-0020166-g001:**
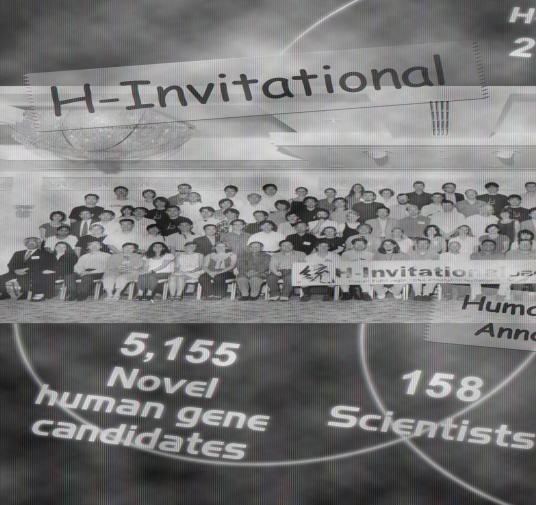
Scientists at the annotation “marathon” of 41,118 cDNA clones

Deciphering the human genome presents such a daunting challenge in part because it's so huge, making it difficult to distinguish genetic signal from noise. Simpler organisms have much more compact genomes. In the case of brewer's yeast, for example, genes that encode proteins account for about 70% of the genome. In contrast, only about 1% to 2% of the human genome codes for proteins. That translates to about one gene for every 2,000 bases for yeast compared to about one gene for every 150,000 bases for humans.

The low density of human genes makes identifying them difficult enough, but this process is further complicated by how genes are organized in the human genome. The functional parts are broken up into smaller segments called exons, which are separated in the genome by intervening sequences called introns. This configuration also occurs in simpler organisms, but since the number and size of introns is relatively small in simpler organisms, it's easier to tell what's a gene and what isn't. In humans, the introns are extremely long, as are the gaps between the genes, and the exons are tiny in comparison; plus, it takes many more of these short, scattered exons to make one gene.

One approach to this problem is to use computer algorithms that scan the genome sequence looking for segments of DNA sequences that could potentially encode proteins. Gojobori and colleagues, however, used a different approach. They analyzed the sequences of 41,118 full-length cDNAs available from six sequencing centers around the world. These cDNAs are stretches of DNA that represent genes that have already been expressed and used by the cell for protein production. Since all the exons have been spliced together and the introns removed, these cDNAs correspond to the functional versions of these genes, allowing researchers to work backward, looking for the sequences in the genome.

In order to process the 41,118 cDNAs, the researchers used a combination of computer algorithms and expert human analysis. To tackle such an enormous project, 158 genome scientists, representing 67 institutions from 12 countries, gathered in Japan in the summer of 2002. Over the course of a ten-day annotation marathon, the scientists validated, mapped, and annotated the cDNAs. As things stand, the team has been able to assemble the cDNAs into over 20,000 strong candidates for human genes.

From just the initial analysis of the data generated by this group, several valuable findings about the human genome have emerged: there are over 5,000 candidates for new genes, including an exciting group of several hundred that do not appear to encode proteins; up to 4% of the genome appears not to be represented in the current human genome sequence; and several thousand DNA sequence variants have been uncovered that will be useful for disease mapping studies.

But perhaps most important of all, the data from this study have been collected and assembled into a large searchable database called H-Invitational Database, which is linked to other functional databases around the world. This will be an invaluable resource for geneticists, and will serve as a starting point for further analyses. Future research on the human genome will be aimed at expanding the list of known genes and analyzing the properties of these genes. This study not only moves us closer to a complete functional description of the human genome, it also builds on the traditions of international cooperation and large-scale collaboration that played such an important part in deciphering the sequence itself.

